# Transfer and contact-induced variation in child Basque

**DOI:** 10.3389/fpsyg.2014.01576

**Published:** 2015-01-21

**Authors:** Jennifer Austin

**Affiliations:** Department of Spanish and Portuguese Studies, Rutgers, The State University of New JerseyNewark, NJ, USA

**Keywords:** bilingualism, language development, language contact, Basque linguistics, Spanish linguistics

## Abstract

Young Basque-speaking children produce Differential Object Marking (DOM) and pre-verbal complementizers in their speech, variants argued to stem from contact with Spanish (Austin, 2006; Rodríguez-Ordóñez, 2013). In this paper, I claim that despite their contact-induced origin, these forms reflect distinct developmental tendencies on the part of the child acquiring Basque. Children's use of pre-verbal complementizers in Basque seems to be a relief strategy that bilingual children employ until they have acquired the post-verbal complementizers in Basque, which are low-frequency morphemes. In contrast, the use of DOM is present in the adult input, although children use this construction to a greater extent than adults do. Finally, I discuss the implications of these findings for the part that child learners play in advancing language change.

## Introduction

Which elements of syntax do bilingual children tend to transfer from one of their languages to the other, and how long do these cross-linguistic effects persist in development? Although bilingual children can differentiate between the grammars of their languages from an early age (Meisel, 1989, 2001; De Houwer, 1990; Paradis and Genesee, 1997), cross-linguistic influence between the grammars of bilingual children has been found in many domains, including the expression of null vs. overt subjects, topic drop, word order, verbal inflection, and the use of determiners (Schlyter, 1994; Gawlitzek-Maiwald and Rosemary, 1996; Döpke, 1998; Yip and Matthews, 2000; Müller and Hulk, 2001; Paradis and Navarro, 2003; Kupisch, 2007). Furthermore, there is strong evidence that the two languages of a bilingual adult are highly interconnected and mutually influence each other during language processing (Kroll et al., 2006, inter alia), which presents the possibility that some cross-linguistic effects seen in early child bilingualism may last into adulthood. In this article, I address this possibility by examining the production of Differential Object Marking (DOM) and pre-verbal complementizers by child and adult Basque speakers, constructions which are considered non-standard in Basque. Specifically, I investigated the degree to which age and bilingualism affected the production of these variants in the speech of monolingual and bilingual children learning Basque.

Previous work found evidence of DOM in Basque-speaking adults (Austin, 2006; Rodríguez-Ordóñez, 2013), and some pre-verbal complementizers in children's speech (Austin, 2001). However, the possibility that pre-verbal complementizers are present in the adult input was not examined in previous research, nor were comparisons made of the production of DOM by children and adults. These questions were investigated in this paper. Before describing the methods used in this study, an overview of research into Spanish-Basque contact phenomena will be provided, followed by a description of DOM and pre-verbal complementizers in Basque.

## Contact between Basque and Spanish

Euskara, the Basque language, is a non-Indo-European language isolate with approximately 800,000 speakers in the Basque Country, a small region between Northern Spain and Southwestern France. In the 20th century, two important demographic changes affected how many people spoke Basque, as well as how proficient they were in speaking the language. First, workers from other parts of Spain moved to cities in the Spanish Basque Country seeking employment, and in doing so nearly quadrupled the population of the region. This influx of workers who were not Basque speakers, combined with Franco's suppression of the minority languages of Spain, turned Basque into a language spoken by only 25% of the population. Another important factor was the loss of Basque monolingualism; presently, all Basque speakers also speak either French or Spanish, with the exception of some young children who have not yet begun formal schooling, such as some of the participants in this study.

The recovery of Basque in the late 20th century was fostered by several developments, principally the end of Franco's dictatorship and the transition to democracy following his death, after which the Basque Country officially became bilingual in 1978. Additionally, in 1982 a new law mandated that all government services be offered in Basque, and also required all public schools to provide access to bilingual education in Basque and Spanish. These measures represented significant progress toward ensuring the survival of Basque because they introduced the language into prestigious spheres of use which had previously been limited to Spanish (Haddican, 2005). Another important factor in the maintenence of Basque was its standardization through the creation of a unified dialect, Euskara Batua (Standard Basque) (Amorrortu, 2000; Haddican, 2005).

Despite centuries of being in contact, the typological characteristics of Basque and Spanish have remained quite different, most notably in their case marking and head direction systems (Silva-Corvalán, 1997). Unlike Spanish, which is a head initial language with nominative/accusative case-marking, Basque has an ergative/absolutive case system and is a head-final language. Nonetheless, the fact that Basque and Spanish have been in contact for well over a 1000 years is readily apparent in both languages. Basque shows evidence of contact with Spanish (and also Latin and French) in its many loanwords and calques. In fact, by one estimate up to 40% of the modern Basque lexicon is comprised of borrowings from these Romance languages^1^.

## Differential object marking (DOM) in Basque^2^

Basque is a triple-agreement language in which the verb is inflected with the person and number features of the subject, indirect object, and direct object. When there is triple agreement on the auxiliary (for the subject, indirect, and direct objects), the absolutive argument (direct object) argument can only be inflected for 3rd person.

A pro-drop language, Basque permits up to three arguments in a clause to be null, as shown in example (1). Most Basque verbs, like the verb in (1) are periphrastic, comprised of a participle and an inflected aux^3^.

(1) *Jarriko    diogu,                 Ø     Ø    Ø             bale?*
      put-FUT ABS3s- DAT-3s- *pro- pro- pro*-ERG ok
                     ERG-1p              ABS DAT
    “(We)'ll put (it) (on him), ok?”

In many languages, case marking is used to differentiate human and non-human direct objects, a pattern known as DOM. This phenomenon occurs in other languages too, such as the use of the “personal a” marker in Spanish that is required with specific, animate direct objects, as seen in (2a,b). DOM also occurs in Hindi, illustrated in example (2c,d) in which the post-position “ko” must be used with human, specific objects.

(2)  Spanish:
       a.         *He          visto        a           mi     hija*
                   have-Isg seen         DOM    my    daughter
                   “I have seen my daughter”
       b.        ^*^He visto mi hija
      Hindi:
      c.          *Aurat    bacce      ko             bula    rahi         hai*.
                   woman  child-      ACC/DAT  call-    PROG     is
                                oblique.sg
                   “The woman is calling the child”
      d.          *?Aurat*   *bacca   bula   rahi     hai*.
                   woman   child      call-   PROG  is
                                                                   Comrie (1989)

In Basque, DOM takes the form of using the dative case to mark animate direct objects in spoken Basque, a pattern which as been argued to result from convergence with Spanish (Austin, 2006; Rodríguez-Ordóñez, 2013). The Spanish spoken in the Basque Country is a dialect with DOM in which dative clitics rather than accusative ones are used to refer to animate direct objects (Fernández-Ordóñez, 1999), as seen in example (3):

(3) *Se      suelta  el   cerdo, el   carnicero le                  agarra*
     REFL release the  pig,     the butcher     DATclitic3Sg grabs
    * de       así*.
     like     this
      “They let the pig go, the butcher grabs it like this”
                                                                           (Landa, 1995)

DOM in spoken Basque can be seen in example (4)a. In this sentence, dative verbal inflection and case are used to mark an animate direct object, instead of the standard absolutive inflection as in (4)b.

(4) *DOM*
      a.     *Nik         zuri      entzun di-zu-t*
              ERG1sg DAT2sg hear    ABS3sg-DAT2sg-ERG1sg
     “I have heard you-DAT”
     Standard Basque
      b.     *Nik          zu         entzun  zaitu-t*
              ERG1sg  ABS2sg hear     ABS2sg-ERG1sg
     “I have heard you-ABS”

The two forms shown in (4) a,b meaning “I have heard you” co-exist in the dialect of Basque spoken in the Spanish Basque Country, but not in the Basque spoken in France, where only the standard form (4)b is used. In both dialects utterance (4)b is grammatical only if there is a previously mentioned, unspecified direct object, meaning something like “I heard it from you.”

A corpus of contemporary adult spoken Basque collected in the Spanish Basque Country contained examples of DOM (Hualde et al., 1994). Similar examples appear in natural speech corpora from children acquiring Basque as an L1 or bilingually (Barreña, 1995; Ezeizabarrena, 1996; Austin, 2001). In these studies, children produced dative case with human direct objects when using verbs such as *jo* “to hit,” *lotu* “to tie up,” and *harrapatu* “to catch.” Barreña notes that examples of DOM are also present in adult speech, and Ezeizabarrena mentions that exchanges such as the one in example (5) are common, in which parents are teachers correct children's use of DOM in Basque:

(5) Child:
      *Ikusi      dotset aitari*
      See        ABS3sg-DAT3sg-ERG1sg father-DAt3sg
      “I have seen (it) on Dad”
      Mother:
      *Zer      ikusi    dotsek                   bada? Belarrixe, ala?*
      what    see      ABS3sg-DAT3sg-  huh      ear            or what
                             ERG2sg
      “What have you seen on him? His ear, or what?”
                                                          Ezeizabarrena (1996)

Austin (2006) argued that DOM in Basque is a contact-induced change that is the result of convergence between Basque and Spanish *leísmo*, or the use of the dative case for animate, specific direct objects. In that paper, I suggested that DOM in Basque is a change in progress that has been accelerated by the bilingualism of Basque speakers, as well as by the large number of L2 speakers of Basque who are dominant in Spanish. Additionally, I suggested that several language-internal factors made the Basque case system susceptible to influence from Spanish in the transfer of DOM, including (1) a diachronic tendency to substitute dative for absolutive agreement in Spanish Basque; (2) language-internal trend toward syncretism or agreement simplification; and (3) the pro-drop nature of Basque, which may lead to reanalysis of agreement morphemes on the part of the learner for sentences such as (6) when it is not clear whether the 3rd person dative argument refers to the indirect or the direct object.

(6) *Lagundu egingo    di-o-t*
      help        do-FUT  ABS3s-DAT-3sg-ERG-1sg
    “I will help him/her (to do it)”> reanalysis:
                                      “I will help him/her”

Rodríguez-Ordóñez (2013) examined the extent to which adult speakers of Gernika Basque found the use of DOM acceptable. Using a grammaticality judgment task, she asked 5 Spanish-dominant and 11 Basque-dominant participants to rate transitive sentences with and without DOM using a Likert scale. She found that both groups of speakers were significantly more likely to rate the non-DOM sentences higher than the DOM sentences. Although the Spanish-dominant bilinguals were more likely than the Basque-dominant ones to accept the DOM sentences, there was not a significant difference in judgments between the two groups. An analysis of verb types found that DOM was significantly more likely to appear with certain verbs (such as *ikutu* ′to touch) than others, and that it was favored in sentences with null dative objects.

In a second experiment, Rodríguez-Ordóñez asked adult speakers of the Gernika dialect of Basque from three different age groups (18–25, 30–45, and 50–65 years old) to rate speech samples from Gernika Basque, Standard Basque and Spanish for “likeability” and “Basqueness.” In each dialect or language, participants heard DOM and non-DOM transitive sentences. Across age groups, sentences from Gernika Basque (with DOM and without) were judged “more Basque” than comparable samples from Standard Basque or Spanish. However, Standard Basque sentences with DOM were judged “less Basque” than non-DOM sentences. In judging “likeability,” an interesting contrast emerged between older and younger participants; whereas the younger speakers judged Gernika Basque samples with DOM to be much less “likeable” than non-DOM samples, older participants did not distinguish between DOM and non-DOM samples from Gernika Basque. Rodríguez-Ordóñez interprets this finding to indicate that younger speakers of Gernika Basque stigmatize DOM to a greater extent than older ones, perhaps due to their receiving literacy instruction in Basque, and having been taught to avoid *erderakadak* or “Spanishisms.” This hypothesis was supported by data that Rodríguez-Ordóñez collected in interviews with adult speakers of Basque, in which she asked them whether using DOM in Basque was acceptable. She found that DOM has highly stigmatized by bilinguals who were Basque-dominant, whereas Spanish-dominant speakers admitted to using DOM in Basque and were more accepting of its use.

## Pre-verbal complementizers in Basque

A second syntactic characteristic which distinguishes Basque from Spanish is the order of constituents in a sentence. Basque is a head-final language in which verbs, complementizers such as relative pronouns, determiners and post-positions always appear on the right side of the phrase or the sentence. For example, Basque sentences have an unmarked subject-object-verb order such as the example in (7):

(7) *Guk         liburu asko irakurri dugu*
      We-ERG book   a lot read       AUX-ABS3sg-ERG1sg
      “We have read a lot of books”

Notice that in addition to the object preceding the verb, the verb precedes the auxiliary. In Spanish, the order of these constituents is the opposite; the direct object follows the verb, and the verb follows the auxiliary, as in (8):

(8) *Nosotros   hemos              leído muchos libros*
      we-NOM have-NOM1sg read   many     books
             ‘’We have read many books”

This pattern of the head of a phrase on the right for Basque and on the left for Spanish is repeated recursively to form all the elements of a sentence. The different position of the inflection node (INFL) in each language reflects the fact that the Basque verb is inflected on the right, and the Spanish one on the left, as seen in examples (9)a, b:

(9) a. Basque:        b. Spanish:
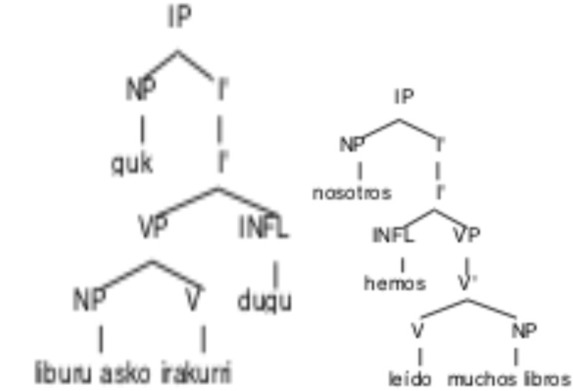

Similarly, embedded clauses in Basque are situated to the left of the embedding element, whereas in Spanish, they are on the right, as shown in (10):

(10) Basque:
        *ikusi  Duen                                     gizon-a*
       seen    AUX-ABS3sgERG3sg RELP man-DET
       “The man that I saw”
       Spanish:
       *el        hombre  que     he                    visto*
       DET   man         RELP AUXNOM1sg seen
       “The man that I saw”

Basque complementizers are also head-final, such as the morphemes -*ela* “that” and -*elako* “because” that attach to the final verb of a clause, as seen in examples (11) a and b:

(11) a. *Gustatzen Zaidelako*
            pleasing    AUX.ABS1sg.DAT.3sg.because.COMP
            “Because I like it”
        b. *Etorriko      zar-ela*
            come.FUT   AUX.ABS2sg.that.COMP
            “That you will come”

In Austin (2001), I found examples of children producing head-initial complementizers in Basque such as (12) and (13)a and b:

(12) a. ^*^zergatik  xx ni    Naiz              txintxua
           why.CO    I.ABS  be.ABS.1sg   good
           MP
          “Because I am good”                                 [XO 3′0]
       b. Target:  *ni         naiz-elako       txintxua*
                       I.ABS  be.ABS.1sg-    good
                                   because.COMP
                       “Because I am good”
(13) a. ^*^ze              ikusi   dut                           zure     ipuina
            that.COMP see     AUX.ERG1sg.ABS  your     book
                                         3sg
            “that I have seen your book”                              [XO 3′0]
      b. Target: *ikusi   dud-ala                           zure     ipuina*
                     see      AUX.ERG1sg.ABS3sg-  your     book
                                COMP
       “That I have seen your book”

I interpreted examples like these to be these to be instances of transfer from Spanish, which has preverbal complementizers, such as those in (14).

(14) a. *Porque              Soy       bueno*
           because.COMP be.1SG good.MASC
           “Because I am good”
      b. *Que           he                   visto tu libro*
          that.COMP  AUXNOM1sg seen
          “That I have seen your book”

However, to prove that these examples stem from cross-linguistic transfer from Spanish, one would need to see whether monolingual children acquiring Basque also produce them, although they are unattested in previous research (Kintana, 2011). If they did, then the production of preverbal complementizers would presumably be a stage that children pass through while learning Basque, rather than the result of bilingual influence from Spanish.

## Research questions

This paper addresses how age and bilingualism affect the production of DOM and preverbal complementizers in Basque-speaking children and adults, phenomena that have been argued to result from contact with Spanish (Austin, 2001, 2006; Rodríguez-Ordóñez, 2013). The research questions under investigation include:

1. Do monolingual children produce preverbal complementizers in Basque?
2. Are preverbal complementizers present in the adult input in Basque?
3. How does the production of DOM by monolingual children, bilingual children and bilingual adult compare?
4. Does early language dominance affect the production of DOM in children, as is the case for bilingual adults (Rodríguez-Ordóñez, 2013)? If so, we would expect bilingual children to produce more utterances with DOM than monolingual ones.

The experimental methods and information about the participants are described in the following section.

## Experimental design

### Adult participants

The adult data reported here come from the analyses on DOM in Basque that were conducted in Austin (2006). In that paper, I categorized the adult participants' degree of proficiency in Basque according to age of acquisition of the language and the participants' own perceptions of their language proficiency. All of the participants were fluent in Basque, and used the language regularly in school or in a professional context. The participants' socioeconomic profiles are provided in Table 1; they were all women, and were bilingual university students from middle-class families. Natural speech transcripts of 2–5.5 h length from each participant were examined and coded for instances of DOM. Their conversational partners were children between 2 and 3.5 years of age (the participants in this study).

**Table 1:** **Adult participants' information (Austin, 2006)**.

**Participants**	**Age**	**City of Residence**	**Language spoken at home**	**Language used in school**
TA	22	San Sebastian	Basque	Basque
BI	20	San Sebastian	Basque	Basque
RS	23	San Sebastian	Spanish	Basque (started learning Basque at age 3)
GF	25	Bilbao	Spanish	Spanish (learned Basque as an adult)

## Child participants

The data for this paper were collected from 20 bilingual children learning Basque and Spanish simultaneously and from 11 monolingual children acquiring Basque. All the participants were living in the Spanish Basque Country at the time of data collection. Children whose parents signed consent forms were selected to participate in the study; the adult participants also signed consent forms. All the consent forms and the study protocol were reviewed and approved by the Cornell University Institutional Review Board. The children who participated in this study spoke Standard (Unified) Basque, but some participants also spoke Bizkaian or Gipuzkoan Basque dialects, and some features from these dialects appeared in their speech. All the bilingual children spoke standard Northern Peninsular Spanish, and were recruited through their schools or through acquaintances of the author. In parental and teacher reports the children were identified as bilingual, simultaneous acquirers of Spanish and Basque. Most children produced many more utterances in Basque than in Spanish. This is most likely due to the fact that most children were interviewed at school, a primarily Basque-speaking environment for many of them, which led them to feel more comfortable speaking Basque there.

The bilingual children who participated in this study were recruited from schools which taught in Basque and Spanish equally (model B) or for which Basque was used exclusively by teachers in talking to children (model D). Table 2 provides information about the children's linguistic backgrounds, including the school models that they attended and their parents' language use.

**Table 2:** **Language background of bilingual child participants**.

**Subject initials**	**Sex**	**Age**	**Input from mother**	**Input from father**	**Input from other family members**	**School model**
GG	M	2;01	Bilingual	Bilingual	No information	B
NI	F	2;01	Bilingual	Bilingual	No information	B
NC	F	2;04	Bilingual	Bilingual	No information	D
LH	M	2;05	Bilingual	Spanish	No information	D
TC	F	2;05	Bilingual	Bilingual	No information	B
AI	F	2;06	Bilingual	Bilingual	No information	D
ME	F	2;06	Basque	Spanish	No information	D
RM	M	2;07	Basque	Spanish	Sister-bilingual	D
IC	M	2;07	Bilingual	Spanish	No information	B
RB	F	2;08	Bilingual	Bilingual	Grandparents-Basque	B
AR	M	2;08	Spanish	Bilingual	No information	B
OH	M	2;08	Bilingual	Bilingual	No information	D
IU	M	2;10	Bilingual	Bilingual	No information	D
XO	M	3;00	Bilingual	Bilingual	No information	D
LA	F	3;00	Basque	Basque	Grandparents-bilingual	D
DG	M	3;01	Bilingual	Bilingual	No information	D
MA	M	3;01	Bilingual	Bilingual	No information	D
IA	M	3;02	Bilingual	Bilingual	No information	B
AM	F	3;03	Basque	Spanish	Siblings-Basque	D
AB	F	3;04	Bilingual	Bilingual	No information	D

The children's ages were between two and three and a half years old. This age range was chosen because it is the point at which verbal inflection and case develop rapidly, and also it allowed for comparison with studies conducted previously (Barreña, 1995; Ezeizabarrena and Larrañaga, 1996).

### Procedures

Speech samples were collected in Bilbao and San Sebastian, as well as in smaller cities in the provinces of Bizkaia and Gipuzkoa. These included Asua, Zeanuri, Hernani, and Oiartzun. One spontaneous speech sample of 30–120 min was recorded while each child interacted with adults (native speaker assistants, the author, or the child's parents) in both languages was collected and transcribed by native speaker assistants, and coded by the author. The native speaker assistants and the parents were all bilingual, and the author is a near-native speaker of Spanish and a non-native speaker of Basque with limited proficiency. At each recording session, there was a bilingual native-speaker adult present who spoke to the children in each language, first one and then the other. If a child did not want to use one of his/her languages, we attempted to collect more data in that language within a few days. If that attempt failed, we did not use the data from that child. The data collection sessions were recorded with an analog tape recorder and the majority were also videotaped. Most of the participants were recorded in their preschools, but some children were also recorded in their homes, in the presence of one or both parents. During the sessions, the children and experimenters read storybooks and/or played games together, including doing puzzles, or playing with dolls, blocks, or puppets. The number of utterances that each child produced in each language is shown in **Tables 5, 6**.

### Child participants

The children's production of verbal agreement and case in Basque finite clauses was analyzed. Children's self-repetitions were excluded from analysis, as were their repetitions of adults speech. Also excluded were utterances with a verb phrase that contained language mixing, as in example (15). In these examples, (B)=Basque, (S)=Spanish.

(15) *Ez         Du                                            le-tzen*
      NEG(B) AUX.ABS.3SG-ERG3SG(B) read (S)- ing(B)
      “He's not reading”                                                      [RM 2;07]

Table 3 provides the age and MLU of the 20 bilingual children who participated in the study.

**Table 3:** **Number of utterances and MLU in each language for bilingual children**.

**Child's initials, sex, age**	**Basque: Number of utterances and MLU**	**Spanish: Number of utterances and MLU**
GG, M, 2;01	283	125
	1.85	1.50
NI, F, 2;01	78	125
	1.18	1.20
NC, F, 2;04	85	17
	2.44	2.26
LH, M, 2;05	71	45
	1.95	2.55
TC, F, 2;05	413	43
	1.53	2.21
AI, F, 2;06	205	110
	2.14	2.38
ME, F, 2;06	200	78
	2.65	2.95
RM, M, 2;07	64	127
	2.20	2.83
IC, M, 2;07	20	148
	2.22	2.95
RB, F, 2:08	364	40
	4.55	5.30
AR, M, 2:08	67	171
	1.85	3.15
OH, M, 2;08	362	65
	3.16	2.69
IU, M, 2:10	126	33
	3.93	5.02
XO, M, 3;00	315	15
	3.16	2.69
LA, F, 3:00	151	83
	4.26	2.53
DG, M, 3:01	67	115
	3.15	4.52
MA, M, 3;01	279	76
	2.48	1.99
IA, M, 3;02	237	168
	2.95	3.83
AM, F, 3;03	115	4
	3.33	2.50
AB, F, 3;04	86	23
	3.24	3.38

MLU was used as an indication of language dominance in Spanish or Basque, together with a language questionnaire and information for the children's teachers. In Table 4 the age and MLU of the monolingual Basque children is shown. As mentioned in the introduction, although there are no longer monolingual adult speakers of Basque because all of them are bilingual, there are some young monolingual Basque-speaking children who speak Basque exclusively at home, and have not yet learned Basque at school.

**Table 4:** **Number of utterances and MLU for monolingual Basque-speaking children**.

**Child's, sex, age**	**Total utterances in sample**	**MLU**
AC, F, 2;01	221	1.95
JH, M, 2;01	126	1.88
MA, M, 2;03	183	1.73
EC, F, 2;05	319	1.65
AH, F, 2;05	162	2.46
MC, M, 2;08	278	1.80
AG, M, 3;00	155	3.24
EG, M, 3;00	117	2.42
ME, M, 3;01	177	3.67
NS, F, 3;02	282	5.07
AB, M, 3;03	257	2.83

The next sections presents the results from the analyses of DOM and preverbal complementizers in child and adult Basque.

## Results

The results from adult speakers of Basque analyzed in Austin (2006) are shown in Table 5. Overall, adult participants produced DOM in 18.3% production of possible contexts with human objects, bilingual children produced DOM in 33% of possible contexts, and monolingual children produced utterances with DOM in 43% of possible contexts.

**Table 5:** **Percentage of adult DOM use in possible contexts (Austin, 2006)**.

**Participants**	**Hours of transcribed natural speech**	**Contexts with human direct objects (transitive verbs)**	**Number of DOM examples found**	**% of DOM in contexts with human direct objects**
TA	5	31	3	9.7
BI	4	10	2	20
RS	5.5	24	6	25
GF	2	6	2	30
Totals	16.5	71	13	18.3

As seen in Table 5, there was individual variation in the use of DOM by the adult speakers.

In natural speech transcripts from 4 adult speakers (9 h total), DOM was produced in 13/71 cases (18%). There were fewer cases of DOM produced by the two adult participants whose first language was Basque than the two participants who had learned Basque as a second language, as seen in Table 4.

Adults used DOM with the following verbs: *entzun* “to hear,” *jo* “to hit,” *jarri* “to put,” *utzi* “to allow” and *molestatu* “to bother.” Examples are provided in (16) a–c.

(16) a. *Eh,    ez      di-zu-t      entzun*
            hey    NEG  ABS3sg-**DAT2sg-ERG1sg** hear-PER
            “I didn't hear you-DAT””                            [TA]
        b. *Ez      dizut                        jarriko      gainean*
            NEG  ABS3sg-**DAT2sg-** put- FUT    on top
                      ERG1sg
            “I won't put you-DAT on top of it”       [BI]
        c. *Zergatik      jotzen      di-    da-zu         neri?*
            Why            hit-IMP    ABS3s-**DAT-**      me-DAT
                                                 1s-ERG-2s
            “Why are you hitting me-DAT?”              [RS]

Table 6 shows the verbs which appeared with DOM in the adults' speech.

**Table 6:** **Verbs used with DOM by adults (Austin, 2006)**.

**Verbs used with DOM**	**Number of examples**
*jarri* “to put”	4
*entzun* “to hear”	4
*jo* “to hit”	2
*utzi* “to allow”	2
*molestatu* “to bother”	1

In most cases, adults' utterances with transitive verbs that take human objects did not trigger DOM; some of the verbs consistently used with standard transitive agreement (non-DOM) included *ikusi* “to see,” *ezagutu* “to know,” *maite* “to love,” *harrapatu* “to catch” and *salbatu* “to rescue.” It seems that animacy triggers the use of Basque DOM, like Spanish leísmo, rather than reflecting a particular verb's subcategorization for a lexically-specified quirky case.

The bilingual children produced utterances in Basque with DOM in 16/48 possible contexts, or 33% of the time on average, as seen in Table 7.

**Table 7:** **Use of DOM in Basque (bilingual children)**.

**Participants' initials, sex, age**	**Basque utterances, MLU**	**Contexts with human direct objects (transitive verbs)**	**Number of DOM examples found**	**% of DOM in contexts with human direct objects**
GG, M, 2;01	283	0		0
	1.85			
NI, F, 2;01	78	0		0
	1.18			
NC, F, 2;04	85	0		0
	2.44			
LH, M, 2;05	71	0		0
	1.95			
TC, F, 2;05	413	2	0	0
	1.53			
AI, F, 2;06	205	2	1	50%
	2.14			
ME, F, 2;06	200	1	1	100%
	2.65			
RM, M, 2;07	64	1	1	100%
	2.20			
IC, M, 2;07	20	0		0
	2.22			
RB, F, 2:08	364	1	0	0
	4.55			
AR, M, 2:08	67	0		0
	1.85			
OH, M, 2;08	362	4	0	0
	3.16			
IU, M, 2:10	126	0		0
	3.93			
XO, M, 3;00	315	17	6	35%
	3.16			
LA, F, 3:00	151	0	0	0
	4.26			
DG, M, 3:01	67	0		0
	3.15			
MA, M, 3;01	279	2	2	100%
	2.48			
IA, M, 3;02	237	10	4	40%
	2.95			
AM, F, 3;03	115	6	1	17%
	3.33			
AB, F, 3;04	86	3	0	0
	3.24			
		48	16	33%

The verbs used by bilingual children with DOM are shown in Table 8.

**Table 8:** **Verbs used with DOM by bilingual children**.

**Verbs used with DOM**	**Number of examples**
*Jan “*to eat”	10
*jarri* “to put”	2
*harrapatu* “to capture”	1
*bota* “to throw”	1
*utzi* “to let”	2

Examples of utterances with DOM produced by bilingual children are provided in (17) a,b.

(17) a. *eta   bota   dio                                             uretara*
            and   throw AUX.ABD3s.DAT3s.ERG3s  water-to
            “and he threw him-DAT in the water”         [IA 3;02]
        b. *Uzten    didazu?*
            let         AUX.ABs3s.DAT1sg.ERG2sg
            “Will you let me-DAT?”                              [MA 3;01]

In Spanish, instances of DOM were considered to be those in which the child used either the differential object marker “a” or a dative clitic with animate, direct objects. Bilingual children began using DOM in Spanish at around 2;06 years, the same time at which it emerged in Basque, as seen in Table 9.

**Table 9:** **Use of DOM in spanish (bilingual children)**.

**Participants' initials, sex, age**	**Spanish uutterances, MLU**	**contexts with human direct objects (transitive verbs)**	**number of DOM examples found**	**% of DOM in contexts with human direct objects**
GG, M, 2;01	125	0	0	0
	1.50			
NI, F, 2;01	125	0	0	0
	1.20			
NC, F, 2;04	17	0	0	0
	2.26			
LH, M, 2;05	45	0	0	0
	2.55			
TC, F, 2;05	43	0	0	0
	2.21			
AI, F, 2;06	110	1	1	100%
	2.38			
ME, F, 2;06	78	0	0	0
	2.95			
RM, M, 2;07	127	1	0	0
	2.83			
IC, M, 2;07	148	0	0	0
	2.95			
RB, F, 2:08	40	0	0	0
	5.30			
AR, M, 2:08	171	1	1	0
	3.15			
OH, M, 2;08	65	0	0	0
	2.69			
IU, M, 2:10	33	0	0	0
	5.02			
XO, M, 3;00	15	0	0	0
	2.69			
LA, F, 3:00	83	2	2	100%
	2.53			
DG, M, 3:01	115	4	4	100%
	4.52			
MA, M, 3;01	76	0	0	0
	1.99			
IA, M, 3;02	168	2	2	100%
	3.83			
AM, F, 3;03	4	0	0	0
	2.50			
AB, F, 3;04	23	0	0	0
	3.38			
		11	10	10/11 91%

An example of the use of DOM in Spanish can be seen in example (18):

(18) *le            quiere              comer*
      him-DAT  want-NOM3sg Eat-INF
      “(he) wants to eat him”     [LA 3′0]

The monolingual children used DOM in 16/37 or 43% of possible contexts, as shown in Table 10.

**Table 10:** **Use of DOM in Basque by monolingual children**.

**Participants' initials, sex, age**	**Basque utterances MLU**	**contexts with human direct objects (transitive verbs)**	**number of DOM examples found**	**% of DOM in contexts with human direct objects**
AC, F, 2;01	221	0		0
	1.95			
JH, M, 2;01	126	0		0
	1.88			
MA, M, 2;03	183	2	0	0
	1.73			
EC, F, 2;05	319	1	0	0
	1.65			
AH, F, 2;05	162	0		0
	2.46			
MC, M, 2;08	278	4	0	0
	1.80			
AG, M, 3;00	155	0		0
	3.24			
EG, M, 3;00	117	7	7	100%
	2.42			
ME, M, 3;01	177	4	2	50%
	3.67			
NS, F, 3;02	282	11	5	45%
	5.07			
AB, M, 3;03	257	11	2	18%
	2.83			
Total		37	16	43%

The verbs used by monolingual children with DOM can be seen in Table 11.

**Table 11:** **Verbs used with DOM by monolingual children**.

**Verbs used with DOM**	**Number of examples**
*Jan “*to eat”	4
*jarri* “to put”	1
*harrapatu* “to capture”	2
*bota* “to throw”	1
*utzi* “to let”	2
*kosk egin “to bite”*	3
*txikitu “to cut”*	2
*jo “to hit”*	1

While the monolingual children used DOM with many of the same verbs as the adults and bilingual children, it is interesting to note that they used DOM with a greater number of different verbs than either of the other participant groups. Some examples of DOM produced by the monolingual children can be seen in (19):

(19) a. *hau kosk egin Dio*
            and bite            AUX.ABD3s.DAT3s.ERG3s
            “This one bit that one-DAT”                            [EG 3;00]
        b. *besteak         harrapatzen                              dio*
            let                  capture-IMP                             AUX
           “Another one captures (him-DAT)”                 [NS 3;02]

Preverbal complementizers in Basque were produced by 4 bilingual children, as seen in Table 12.

**Table 12:** **Use of preverbal complementizers (bilingual children)**.

**Participants' initials, sex, age**	**Basque utterances, MLU**	**Preverbal complementizers**	**Post-verbal complementizers**
GG, M, 2;01	283	0	0
	1.85		
NI, F, 2;01	78	0	0
	1.18		
NC, F, 2;04	85	0	0
	2.44		
LH, M, 2;05	71	0	0
	1.95		
TC, F, 2;05	413	0	0
	1.53		
AI, F, 2;06	205	0	0
	2.14		
ME, F, 2;06	200	0	0
	2.65		
RM, M, 2;07	64	0	0
	2.20		
IC, M, 2;07	20	0	0
	2.22		
RB, F, 2:08	364	7	0
	4.55		
AR, M, 2:08	67	0	0
	1.85		
OH, M, 2;08	362	0	0
	3.16		
IU, M, 2:10	126	1	0
	3.93		
XO, M, 3;00	315	1	0
	3.16		
LA, F, 3:00	151	0	0
	4.26		
DG, M, 3:01	67	0	0
	3.15		
MA, M, 3;01	279	0	0
	2.48		
IA, M, 3;02	237	5	1
	2.95		
AM, F, 3;03	115	0	2
	3.33		
AB, F, 3;04	86	0	0
	3.24		
Total		14	3

Preverbal complemetizers were used by 4 children between the ages of 2;08 and 3;02, as shown in the following examples in (20) and (21) (non-target utterances are marked with an asterisk):

(20) a. ^*^ze        dako (dakot)                        mokoa
             COMP have.ABS3s.ERG3sg          mucus
             “That I have a runny nose”               [IU, 2′10]
        b. Target:  *Dakod-ala                      mokoa*
                          have.ABS3s.ERG1sg.COMP mucus
            “That I have a runny nose”
(21) a. ^*^ze                etorri  da                               G. ta Jennifer
             that.COMP  come  AUX.ABS3sg           G and Jennifer
             “that G. and Jennifer have come”              [RB 2;08]
        b. Target:       *etorri  direla                           G. ta Jennifer*
                              come   AUX.ABS3pl-COMP  G. and Jennifer
            “that G. and Jennifer have come”

One child, (IA, 3;02) was able to use a target-like post-verbal morpheme for one complementizer (*ela* “that”) as seen in (22)a, but used a non-target preverbal complementizer for -*elako* “because,” as shown in (22)b.

(22) a. *Jan jan        di- dio-la*
            Eat eat          AUX-ERG3sg-DAT3sg-
                                 ABS3sg-that
            “That s/he ate him/her”            [IA 3;02]
        b. ^*^zergatik        badoa                      eskuelara
             Why-COMP  go.ABs3sg               school-to
               “Because s/he goes to school” [IA 3;02]

All the adults and the monolingual Basque-speaking children used standard post-verbal complementizers exclusively, as seen in example (23).

(23) *Gertatu  zaio-lako*
         happen   AUX.ABS3sg.DAT3sg-COMP
      “Because it happened to him/her”             [ME 3′1]

These examples were rare in the adult input. In 1488 utterances containing clauses from Basque-speaking adults, I found 7 uses of the -*elako* “because” morpheme, and 8 examples of the *-ela* “that” complementizer.

In Spanish, pre-verbal complementizers were produced by adults and two of the bilingual children (AR and IA), whose utterances with preverbal complentizers can be seen in (24):

(24) a. *también  que       se         le              cae          una   gota*
            also         COMP  REFL  DAT.3SG  Fall.2SG a        drop
            “Also, that a drop fell on him”                               [IA 3;02]
        b. *un  moto            que               va          a  lagua*
                  *(al agua)*
            a     motorcycle that-COMP Go.3SG to the water
            “A   motorcycle that goes to the water”  [AR 2;08]

## Discussion/conclusions

While there is good reason to think that both DOM and pre-verbal complementizers in Basque are morphosyntactic outcomes of contact with Spanish, children's production of the two phenomena is quite different, in part reflecting their presence or absence in the adult input. DOM is used by adults as well as children, supporting the hypothesis that it is a change in progress occurring in Basque which results from morphosyntactic convergence with Spanish (Austin, 2006), consistent with the predictions of Sánchez's Functional Convergence Hypothesis (2003, 2004). The finding that DOM is produced more by Spanish-dominant bilingual adults and that those bilinguals find its use more acceptable than Basque-dominant ones is also consistent with this analysis (Rodríguez-Ordóñez, 2013).

Monolingual children produced utterances with DOM to a greater degree (43%) than bilingual children (33%) or adults (18%), suggesting the possibility that both groups of children are not merely matching the patterns present in the adult input. Rather, their behavior may reflect a tendency to generalize or regularize inconsistent patterns, as has been found to be the case for children acquiring an artificial grammar (Kam and Newport, 2005). Of the monolinguals, 4/11 children over the age of 3:00 used DOM in Basque, compared to 7/20 bilinguals. The bilingual children began using DOM earlier, at age 2;06, the same age at which DOM emerged in their Spanish. Because the number of productions of DOM in this study is quite small, more data would have to be collected to evaluate this hypothesis more carefully. It is possible, for example, that bilingual and monolingual children receive input in Basque that differs with respect to the presence of DOM; this could be true, for instance, if bilingual children receive greater exposure to Basque at school than home, and teachers at school use less DOM than the parents of monolingual Basque-speaking children do^4^.

While the finding that monolingual children produced more DOM than bilingual children was unexpected, a Chi-square test indicated that this difference between the groups of children was not significant. The monolingual children's greater use of DOM was a surprise, given than use of DOM is correlated with dominance in Spanish in bilingual adults. Perhaps monolingual children's use of DOM reflects that they are more sensitive to case-marking as a syntactic cue in Basque than bilingual children; exploiting case as a cue in understanding grammatical relations has been argued to develop over time in multilingual children acquiring Light Warlpiri and Lajamanu Warlpiri, for example (O'Shannessy, 2011). Of the 7 children who produced DOM in Basque, 6 attended Basque-only schools (model D, as seen in Table 2), suggesting that for children, more exposure to Basque may correlate with greater use of DOM.

The use of pre-verbal complementizers presents a very different developmental pattern. These forms are used exclusively by four bilingual children between the ages 2;08 and 3;02, and were never produced by monolingual children or adults. Five bilingual children in this age range never used them at all, and their production does not seem to be correlated with their MLU in Basque. I interpret this variability in their use to indicate that children may use pre-verbal complementizers in Basque as a relief strategy until they have acquired the post-verbal complementizers in Basque, which are low-frequency morphemes. However, the variability in the use of pre-verbal complementizers between children suggests that the transfer of this feature from Spanish to Basque is not a universal stage in bilingual Basque/Spanish development. Furthermore, these preverbal complementizers do not seem to reflect a more fundamental type of cross-linguistic influence such as the wholesale transfer of the head-initial properties from Spanish to Basque, since bilingual children's grammars in other ways are consistent with the head-final characteristics of Basque. Rather, I understand these utterances to be a type of temporary relief strategy which may be used by some bilingual children when they are confronted with a construction that they have not yet acquired, following proposals by Gawlitzek-Maiwald and Rosemary (1996) and Bernardini and Schlyter (2004). In Gawlitzek-Maiwald and Rosemary (1996) longitudinal study of a bilingual child learning German and English, they proposed that the child was transferring a German IP to English because she was more grammatically advanced in German than English, as seen in example (25):

(25) ^*^Ich hab gemade you much better.
         “I have made you much better”
                [Gawlitzek-Maiwald and Rosemary, 1996, p. 914]

The child produced mixed-IP utterances such as the one in (26) until she was 2;09 years, at which point the authors claimed she acquired an IP in English too. Gawlitzek-Maiwald and Rosemary referred to the use of the L1 functors in the L2 as *bilingual bootstrapping*, which they argued was a kind of relief strategy for filling in syntactic gaps in the bilingual child's weaker language. Bernardini and Schlyter (2004) came up with a similar proposal, the *Ivy Hypothesis*, in which they suggested that child bilinguals can use their stronger language as a kind of syntactic scaffolding for the weaker one. They also claimed that syntactic transfer from the stronger to the weaker language is possible in all areas of grammar.

The results from this study, in particular, the finding that preverbal complementizers are only used by bilingual children as well as the fact that they disappear as target-like post-verbal forms begin to be produced supports the hypothesis that preverbal complementizers are used temporarily in bilingual development as a relief strategy. However, while the result from this study are suggestive, there are only a few data points from a few children with regard to pre-verbal complementizers (which were only used by 4 out of 20 bilingual children and none of the monolingual children). Longitudinal data would be needed to determine whether the use of pre-verbal complementizers in Basque is in fact a type of short-term transfer similar to the temporary cross-linguistic influence that has been found in bilingual children acquiring many different language pairs (Döpke, 1998; Yip and Matthews, 2000; Müller and Hulk, 2001; Paradis and Navarro, 2003, inter alia). In contrast, the use of DOM seems to be a change in progress that is spreading through the Basque language, as evidenced by the finding that both monolingual and bilingual children use DOM more often than adults and with a greater range of verbs. It is possible that if adult speech is inconsistent with regard to the use of DOM in verbs such as *ulertu* “to understand” that children may adopt a strategy of agreement simplification, such as using dative agreement with all human direct objects, in line with the findings of Kam and Newport (2005). In this regard, child learners could play as important a role as second language speakers of Basque in promoting DOM.

### Conflict of interest statement

The author declares that the research was conducted in the absence of any commercial or financial relationships that could be construed as a potential conflict of interest.

## Footnotes

^1^Bakker (1989, p. 117).

^2^In this paper, I will be looking at the dialects of Standard Basque spoken in the Spanish Basque Country.

^3^Basque is considered a morphologically ergative language. In the gloss in example in (1), ABS, absolutive case; DAT, Dative; ERG, Ergative.

^4^This possibility was suggested to me by an anonymous reviewer.
